# Progressive Muscle Relaxation as an Alternative Therapy for Death Anxiety and Sleep Quality in End‐Stage Cancer Patients: An Experimental Study

**DOI:** 10.1002/hsr2.72140

**Published:** 2026-05-05

**Authors:** Hadi Hasani, Majid Kazemi, Seyed‐Mohammad‐Ebrahim Pourhosseini

**Affiliations:** ^1^ Department of Medical Surgical Nursing School of Nursing and Midwifery, Rafsanjan University of Medical Sciences Rafsanjan Iran; ^2^ Department of Medical Surgical Nursing, Faculty of Nursing and Midwifery, Non‐Communicable Disease Research Center Rafsanjan University of Medical Sciences Rafsanjan Iran; ^3^ Department of Oncology, Faculty of Medicine Rafsanjan University of Medical Sciences Rafsanjan Iran

**Keywords:** cancer, death anxiety, neoplasm, progressive muscle relaxation, sleep quality

## Abstract

**Background and Aims:**

Progressive muscle relaxation (PMR) is a complementary therapy to reduce tension. The present research was conducted to assess the effect of the Progressive muscle relaxation (PMR) on death anxiety and sleep quality of cancer patients.

**Methods:**

This was an experimental study with pre‐test and post‐test design. 60 cancer patients were selected randomly and randomly assigned experiment or control group. Death anxiety and sleep quality of samples were assessed by Templar's Death Anxiety Scale (DAS) and Pittsburgh Sleep Quality Index (PSQI). PMR was trained to patients in eight sessions during 1 month. Data were analyzed using descriptive and inferential statistics (Chi‐squared, Fisher's exact test, independent sample *t*‐test, and analysis of covariance).

**Results:**

A significant decrease in death anxiety and sleep quality scores in the intervention group (*p* < 0.001). The only sleep quality dimension that didn't change meaningfully in the intervention group was the use of sleep medications.

**Conclusions:**

PMR is a method that can be used to reduce death anxiety and improve sleep quality in cancer patients.

## 1 Introduction

Cancer is a devastating disease characterized by abnormal cell growth and uncontrolled division, forming tumors that can invade surrounding tissues and spread to other parts of the body [1, 2]. Indubitably, it is one of the leading causes of today's high mortality rate due to chronic diseases. In 2020, about 19.3 million new cases were diagnosed worldwide, and almost 10 million cancer deaths occurred [3], a figure that highlights the severity of the global cancer burden as the disease has become even more prevalent in recent years [4]. The World Health Organization (WHO) estimated that between 2019 and 2020, cancer was the first or second cause of death among the population under 70‐year‐old [5].

Cancer patients suffer from numerous physical complications such as pain, cachexia, hyperglycemia, coagulation disorders, etc. Inui [6]. They also struggle with various psychological disorders, including body image disorder, sleep disorders, depression, decreased quality of life, suicidal ideation, sexual dysfunction, fear of cancer recurrence, and death anxiety [7].

Death anxiety is a complex concept, referring to the fear associated with mortality awareness [8, 9, 10].

Most of the patients experience a sense of tension and anxiety concerning the thoughts of imminent death following the diagnosis of cancer [11]. Previous studies in Iran reported that 59.91% of Iranian cancer patients had death anxiety [12]. This death anxiety can negatively affect both the patient and their caregivers, interfere with the treatment process, and prevent the patients from adjusting to their current situation [13].

Death is both a biological and psychological phenomenon, and individuals' perceptions of death are shaped by their socialization experiences [14]. In addition, each patient may choose a different Emotion Cognitive Regulation Strategy when he/she faces cancer, and this can impact the level of anxiety he/she experiences. For instance, individuals who use some negative strategies like Avoidance, Suppression, Rumination, Self‐Blame, or Other‐Blame have more anxiety than those who use positive Strategies such as Acceptance, Positive Reappraisal, Self‐ Positive Refocus [15]. Moreover, some factors like gender, education, social class, being in the middle ages, belief in the supernatural, quality of life, and attitudes of caregivers are shown to be contributory factors in the level of death anxiety experienced by the patients [16, 17, 18, 19]. This anxiety is more about the process of a dreadful dying, rather than the death itself. Thus, palliative measures can be a great relief for these patients [13].

One of the other major comorbidities in cancer is sleep alterations. It's estimated that about 30% to 75% of cancer patients suffer from sleep disturbances [20], and up to 95% after 10 years of survivorship [21]. Some causes of these sleep disturbances are due to physical complications such as pain, fatigue, chemotherapy‐induced nausea, leg restlessness, sleep‐related breathing disorder (SRBD), obstructive sleep apnea syndrome (OSAS) [21], and some are psychological like depressive feelings, variable mood, recurring thoughts, and concerns about the disease, bad dreams, cancer‐related fears and anxiety [22, 23]. Up to 90% of cancer patients experience cancer‐related fatigue (CRF) [24], which can lead to sleep disturbance [25], and these sleep problems can interfere with the treatment regimen, cause poor quality of life, reduce treatment follow‐up, and ultimately even increase the risk of death [26].

There are various ways to heal anxiety and sleep disorders in cancer patients. One of the routine ways is pharmacological treatment, which is often considered the first‐line intervention in hospitals. It's estimated that around 28%–40% of cancer patients and cancer survivors use sleep medications [27] such as Zolpidem, Chlordiazepoxide, Opioids, etc., for treating their sleep or anxiety problems [28]. Although pharmacological treatment, especially the use of sleep medications, is common in this context, these drugs not only impose financial burdens and pose serious side effects such as kidney and liver damage [29], but also fail to address many of the physical and psychological causes of sleep disturbances [30]. Moreover, the personal and multidimensional nature of sleep and anxiety problems for each individual can reduce patient satisfaction with these medications [24, 31].

For the aforementioned reasons, complementary therapies such as yoga, spiritual care, guided imagery, music therapy, acupressure, transcutaneous electrical acupoint stimulation, breathing exercise, cognitive‐behavioral intervention, hope Therapy, etc., are often suggested for these patients [25, 32, 33]. Among them, Progressive Muscle Relaxation (PMR) is a simple and low‐cost technique that, without any side effects and without depending on things or subjects outside of oneself, can help patients reduce their anxiety and disturbances [34]. PMR refers to contracting and relaxing all muscle groups one by one, and thinking about free and deep breathing in the middle of this exercise. Potential mechanisms may include modulation of autonomic nervous system activity (e.g., increased parasympathetic tone, reduced sympathetic activation) or neurobiological changes (e.g., reduced cortisol levels) [35, 36, 37].

Recent works suggested that PMR has a positive effect on pain in cancer patients [32] and may reduce the use of consumption of psychotropic and analgesic drugs in these patients [38], but few studies use this technique for patients who have high death anxiety or poor quality of sleep. Given the limited number of studies in this area, this study aimed to assess the effectiveness of PMR on death anxiety and sleep quality of cancer patients.

## 2 Material and Method

### 2.1 Study Design

This interventional study was undertaken in a repeated‐measures design with two time points from March 5, 2018, to December 2, 2023.

### 2.2 Participants

A total of 60 subjects with cancer (Stage III and IV) were recruited from the Ali‐Ibn Abi‐Talib Hospital and Rafsanjan Oncology Clinic (Rafsanjan, Iran). The patients who had a definite cancer diagnosis and were alerted about their diagnosis at least 1 day after starting the treatment were referred to the researcher by the oncologist. Before any further actions, the researcher filled patients with information about the purpose and structure of the study and obtained informed consent from them. Then, the patients who met the inclusion criteria were randomly assigned to intervention and control groups using block randomization. Random allocation sequences were generated using SPSS software. The random allocation sequence was securely maintained as confidential within opaque sealed envelopes, and it remained undisclosed to individuals engaged in data collection, participant recruitment, and the statistical consultant. The personal data of patients, including their names and diagnoses, was considered highly confidential, and the patients were free to leave the study at any point they desired. While the control group continued to receive routine treatments and care, the intervention group started to practice the PMR technique.

The inclusion criteria included the following: (1) having some kind of cancer diagnosis and being alert of that at least for 1 day after stating the chemotherapy/radiotherapy treatment, (2) being interested in participating in the study, (3) being at least over 18, (4) getting 7 scores or above in Templar's Death Anxiety Scale (DAS) which shows at least moderate anxiety, (5) getting 6 scores or above in Pittsburgh Sleep Quality Index (PSQI) which shows sleep impairment (6) having no history of mental disorders, neurological impairment, limb paralysis, or limb amputation, (7) having no bruise or wound in limbs, severe pain or DVT in limbs, (8) not participating in Relaxation classes before, nor doing this technique before [39], (9) having full awareness and acceptable listening and speaking ability to learn relaxation method [40], (10) getting 20 scores or more in Karnofsky Performance Scale [32], (11) having no psychological trauma in the last 6 month, 13) being aware of his/her cancer diagnosis at least for 1 day.

The exclusion criteria were (1) wanting to leave the study, (2) not doing PMR more than two sessions, (3) death, (4) deteriorating patient's condition in a way he/she couldn't cooperate, (5) taking place of an unanticipated happening that causes more anxiety for the patient during the study, (6) getting cranial radiation therapy (because some studies suggested that there is an association between cranial radiation and hypersomnolence) [41].

### 2.3 Sample Size

Using a previous study [42], and comparing the means and standard deviations between the pre and post data reported in that article, and with the help of the formula below, the sample size in each group was calculated as 26.7, and considering 20% expected loss rate, the final sample size in each group considered 30 patients. (Significance level (α): 0.05; Power level: 90% (0.90))

$$n=\frac{(s_{1}^{2}+s_{2}^{2}){\left(z_{1-\frac{\alpha }{2}}+z_{1-\beta }\right)}^{2}}{{\left({\bar{x}}_{1}-{\bar{x}}_{2}\right)}^{2}}=\frac{{\left(1.96+1.28\right)}^{2}\left(0.41^{2}+0.53^{2}\right)}{{\left(0.42\right)}^{2}}=26.7$$

### 2.4 Blinding

In this study, due to the nature of the intervention, patients could not be blinded. However, the statistical analyst and data collector were unaware of the allocation of individuals to the intervention and control groups.

### 2.5 Study Instruments

This study used three measurement tools, i.e., a demographic questionnaire, Templar's Death Anxiety Scale (DAS), and Pittsburgh Sleep Quality Index (PSQI).

The demographic questionnaire assessed the gender, age, education level, marital status, monthly income, the type of cancer, and the stage of cancer. If the patients weren't sure about the exact medical terms of their diagnosis and the stage of cancer, then the researcher used the information obtained from the physician.

The participants' sleep quality was assessed using PSQI, which is a standard self‐report questionnaire designed by Buyssue et al. in 1989 to determine the quality of sleep. This questionnaire measures sleep quality during the past month and includes nineteen questions in seven dimensions (subjective sleep quality, sleep latency, sleep duration, habitual sleep efficiency, sleep disturbances, use of sleep medications, and daytime dysfunction). The sum of these seven dimensions becomes the overall sleep quality, which is a score between 0 and 21. A score of five or more shows that the person has a sleep problem, and as the score goes up, the quality of sleep becomes poorer. This scale has been used in many studies that spell out its high validity and reliability, and studies have shown concordant results of this questionnaire with polysomnography examinations [43]. Psychometric properties of the Persian version of this questionnaire have been approved for the Iranian population. Saeedi et al proved the content validity of the Persian version of this questionnaire by asking eleven experts. They also proved its reliability by means of internal consistency and test‐retest with a correlation coefficient of 0.78 and a Cronbach's alpha of 0.85 [40]. Other studies in Iran reported Cronbach's alpha of 0.79 [44] and 0.87 [45], which reveals the great acceptability of this index in Iran.

Templar's Death Anxiety Scale (DAS) was another Scale that was used in our study, which consists of 15 yes–no questions. The answer YES to each question gets one point, and the answer NO gets zero points. The total score range is between 0 and 15, and higher scores reveal more death anxiety. This score categorized the death anxiety as Mild (0–6), Moderate [10, 43], and High [33, 46]. The validity and reliability of this questionnaire are well established in different studies [47]. The Persian translation is also proven to be applicable to the Iranian community with the split‐half reliability coefficient of 0.62, and Cronbach's Alpha coefficient of 0.73 [48].

### 2.6 Intervention

After obtaining the required permissions, intervention sessions based on PMR were held at the Ali‐Ibn Abi‐Talib Hospital, and Rafsanjan Oncology Clinic (Rafsanjan, Iran). The researcher instructed the patients of the intervention group individually, and he wanted the patients to do PMR (according to Jacobson's relaxation technique) for the next month twice a week, each time for 20 min. During teaching PMR, the patients were provided a calm environment without any disturbance and asked to first take a few deep breaths and then tighten and relax specific muscle groups in order from the upper body limbs down to the legs. They were asked to tense the muscles tightly for 5 s and then relax them for 10 s and think about the difference in feeling between a tense and a relaxed muscle. The preferred position for relaxation was either supine or sitting down [49].

The first session was performed by the researcher, and the patients were asked to do the procedure along with the researcher. The remaining sessions were performed with the help of the caregivers and patients, and at least at the end of every week, the patients were followed up to make sure they did the PMR right and on time.

The control group was also randomly allocated and matched with the intervention group in terms of gender and age. The routine care and treatment of the oncology ward and clinic were implemented on them, and they filled out the questionnaires at the start of the study and 1 month later.

**Figure 1:**
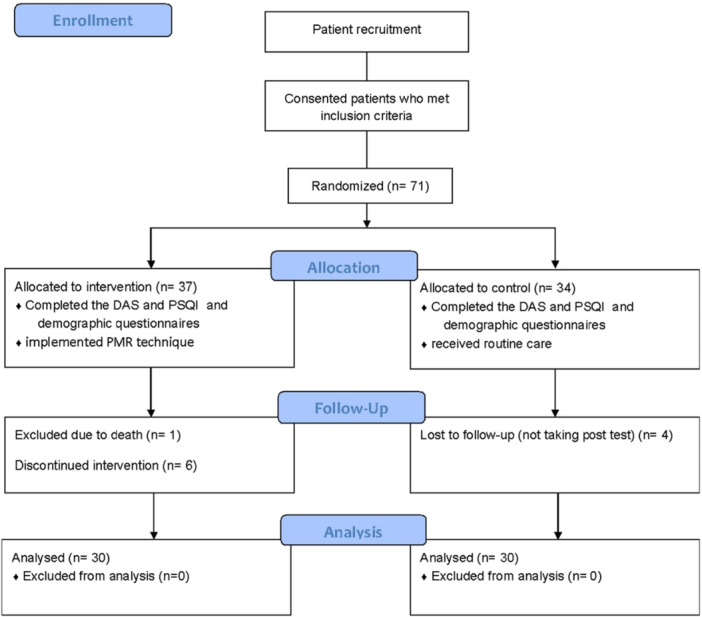
The CONSORT flow diagram.

During the data collection phase, the researcher went into the study setting daily and found the participants who were admitted again to continue their chemotherapy or treatments, and followed them up. In case the patients didn't return to the ward or clinic for 1 week, they followed up via phone call. After 1 month, each patient filled the questionnaires again, and the data were gathered (Figure 1).

### 2.7 Data Analysis

The information obtained from questionnaires was entered into SPSS software, Version 26. The significance level was set at *p* < 0.05 with a 95% confidence interval. It is worth mentioning that the 2‐sided *P*‐value was used for the conclusion as the nature of the hypotheses was 2‐sided. We also followed SAMPL and CONSORT guidelines to report the data and study results [50, 51].

The normality of quantitative variables was assessed using the Kolmogorov–Smirnov test. For normally distributed variables, descriptive statistics (mean ± standard deviation) were reported. For categorical variables, frequencies and percentages were presented. For comparing the demographic variables between the two groups, the Independent Samples *t*‐test, Chi‐square test, and Fisher's Exact Test were used.

For analyzing the pre and post‐scores of sleep quality and death anxiety within the intervention and control group, the paired samples *t*‐test was used, and the mean score of each variable was compared before and after the intervention. The independent samples *t*‐test was used to compare the death anxiety and sleep quality between the two groups.

Also, the Analysis of Covariance (ANCOVA) test was used to check whether the pre‐test differences in death anxiety score between the intervention and control group had an effect on the post‐intervention results. Before using the ANCOVA test, we checked the basic requirements and assumptions of the ANCOVA test. Therefore, we ensured that the data had a normal distribution, significantly correlated using the Pearson test, and the covariate variable did not show a significant relationship between control and intervention group using an independent sample *t*‐test, the homogeneity of variances using the Levene's test, and the homogeneity of regression slopes using a univariate linear model (*p* = 0.565).

### 2.8 Ethics Approval

This study was approved by the Ethics Committee of Rafsanjan University of Medical Sciences (approval code: IR. RUMS. REC.1400.175). All procedures involving human participants were performed in accordance with the ethical standards of the national research committee and with the 1964 Helsinki Declaration and its later amendments or comparable ethical standards. Written informed consent was obtained from all participants and their families prior to inclusion in the study, both verbally and in writing. Participants were assured of the confidentiality of their information and were informed that they could withdraw from the study at any time without any consequences.

## 3 Results

In this study, the mean age (±SD) of the intervention group was 57.63 ± 15.176, and the mean age of the control group was 60.37 ± 14.836 in which the Independent Samples *t*‐test showed no statistically meaningful difference between the two groups (mean difference = −2.74 years, 95% CI: −10.48 to 5.00, *p* = 0.841). The Chi‐Square Test showed that there was not a statistical significance in terms of gender in the two groups (*p* = 0.121). The Chi‐Square Test results also revealed that the two groups didn't differ significantly in either marital status (*p* = 0.389), financial status (*p* = 0.283), or education level (*p* = 0.711).

Using Fisher's Exact Test, we concluded that the difference in the type and the stage of cancer in the two groups is not statistically meaningful (Table 1); also, the stage of cancer for most of the patients was stage 4 (Table 2).

**Table 1:** The type of cancer in the intervention and control group participants.

Variable	Intervention group	Control group	*p* value
Hematopoietic cancers	7 (23.33%)	4 (13.33%)	1.000
Gastrointestinal cancers	8 (26.67%)	6 (20%)	
Genitourology cancers	4 (13.33%)	5 (16.67%)	
Lung and laryngeal cancers	6 (20%)	5 (16.67%)	
Breast cancers	5 (16.67%)	10 (33.33%)	

**Table 2:** The stages of cancer in intervention and control group participants.

Cancer stages	Intervention group	Control group	*p* value
Stage 2	1 (3.3%)	2 (6.7%)	0.506
Stage 3	1 (3.3%)	3 (10%)	
Stage 4	28 (93.3%)	25 (83.3%)	

For analyzing sleep quality and death anxiety variables, we used parametric tests as the variable had a normal distribution. For comparing these variables before stating the intervention between the two groups, we used an independent *t*‐test. The results of these tests showed that the sleep quality of patients entered into two groups was not statistically different before starting the intervention, but the intervention group had more death anxiety (*p* = 0.721, 0.006, respectively) (Table 3).

**Table 3:** The mean score of sleep quality and death anxiety before stating the intervention.

Variable		Mean ± SD	*p* value
Total sleep quality score	Intervention group	13.300 ± 3.544	0.721
	Control group	9.400 ± 3.700	
Death anxiety score	Intervention group	9.566 ± 2.254	0.006
	Control group	8.233 ± 1.406	

Paired samples t‐test results showed that death anxiety and all aspects of sleep quality scores after implementing the PMR meaningfully decreased, except “the usage of sleeping medication” in the intervention group, but no meaningful difference was observed in the control group after the intervention (Table 4). Many participants were already taking sleep medications regularly, which may have limited the potential for change in this subscale.

**Table 4:** The mean score of sleep quality variables and death anxiety of the two groups before and after the intervention.

Variable		Pre‐test	Post‐test	*p* value
Subjective sleep quality	Intervention group	1.700 ± 0.794	1.333 ± 0.922	0.014
	Control group	1.500 ± 0.682	1.633 ± 0.850	0.255
Sleep latency	Intervention group	2.233 ± 1.040	1.833 ± 0.912	0.026
	Control group	2.066 ± 0.907	1.933 ± 0.944	0.211
Sleep duration	Intervention group	2.133 ± 1.041	1.800 ± 0.886	0.010
	Control group	1.200 ± 0.961	1.300 ± 0.987	0.184
Habitual sleep efficiency	Intervention group	2.100 ± 1.155	1.600 ± 1.037	< 0.001
	Control group	0.733 ± 1.048	0.866 ± 1.105	0.103
Sleep disturbances	Intervention group	1.633 ± 0.668	1.300 ± 0.794	0.002
	Control group	1.533 ± 0.776	1.500 ± 0.820	0.769
Use of sleeping medication	Intervention group	1.100 ± 1.398	1.066 ± 1.362	0.326
	Control group	0.666 ± 1.022	0.700 ± 1.118	0.326
Daytime dysfunction	Intervention group	2.366 ± 0.718	1.900 ± 0.711	0.002
	Control group	1.700 ± 1.022	1.800 ± 1.095	0.326
Total sleep quality score	Intervention group	13.300 ± 3.544	10.866 ± 3.766	< 0.001
	Control group	9.400 ± 3.700	9.966 ± 4.122	0.207
Death anxiety score	Intervention group	9.566 ± 2.254	7.733 ± 2.531	< 0.001
	Control group	8.233 ± 1.406	8.133 ± 1.775	0.698

We also conducted a paired t‐test on the difference in scores of each individual between pre and post intervention scores to ensure that the decrease in the death anxiety and sleep quality was statistically meaningful. In this term, the P‐value of the t‐test related to the difference in scores of death anxiety and sleep quality in intervention and control groups was < 0.001 and < 0.001, respectively, which shows that the decrease in the score of the intervention group was statically meaningful in comparison to the control group.

The results of the ANCOVA test showed that there was a statistically significant difference in the death anxiety scores after the intervention between the two groups (*p* = 0.009) (Table 5).

**Table 5:** Results of the ANCOVA test for the effect of pre‐death anxiety scores on post‐death anxiety scores.

Death anxiety	Intervention group Mean ± SD		Control group Mean ± SD			*p* value
Group	Before	After	Before	After	F	
	9.566 ± 2.254	7.733 ± 2.531	8.233 ± 1.406	8.133 ± 1.775	7.243	0.009

## 4 Discussion

The first objective of this study was to investigate the effect of PMR on death anxiety in cancer patients. The results revealed that PMR is an effective technique to reduce death anxiety in cancer patients. In reviewing the literature, no study was found about the effect of PMR on death anxiety, but previous studies found that the PMR technique is an effective way for reducing other types of anxiety, such as prenatal anxiety [52], anxiety in burn patients [53], nurses' anxiety [54], etc. On the other hand, some studies found that PMR is not effective in reducing stress [55]. These inconsistencies may be due to differences in study design, patient characteristics, intervention protocols (frequency and duration of PMR sessions), and the outcome measures used. Furthermore, adherence to the PMR protocol and the presence of additional psychological or medical interventions may also influence the results. PMR tends to be most effective for symptoms closely related to psychological stress, such as anxiety and sleep disturbances, while its impact on broader outcomes like overall quality of life may be less pronounced. It is worth mentioning that some other studies that worked on other relaxation techniques found these techniques ineffective in reducing anxiety. For example, Kurniasari didn't get a positive result out of the Benson technique for reducing anxiety among hemodialysis patients [56], and Calisi didn't conclude that the Benson relaxation technique is useful for reducing nurses' level of anxiety [46]. So, there is a need for more studies on the Effect of the PMR technique introduced by Jacobson on death anxiety to be sure of the efficacy of this intervention among cancer patients.

The second objective of ours was to investigate the effect of PMR on sleep quality in cancer patients. The results showed that PMR is effective for improving the sleep quality of cancer patients, which is aligned with the results of the study of Atadokht et al [39]. The PMR in the present study improved all dimensions of sleep quality except the use of sleep medications, which is also agreed by other studies [40]. PMR was also found to be effective in improving sleep quality in children with COVID‐19 [57] and patients with rheumatoid arthritis [58]. The studies that used the PMR on cancer patients also reported that this technique can reduce the pain, use of analgesic drugs, and nausea in these patients [32, 38, 59].

The mechanism behind PMR's effects is believed to involve modulation of the autonomic nervous system, leading to reduced sympathetic activity and increased parasympathetic tone, which lowers physiological arousal and stress. PMR may also decrease cortisol levels and enhance patients' sense of control and self‐efficacy, thereby improving their ability to cope with cancer‐related psychological distress.

## 5 Conclusion

According to the results presented in this study, progressive muscle relaxation has a positive effect in ameliorating death anxiety and enhancing the sleep quality of cancer patients as well. Due to that, it can be considered as a complementary therapy for cancer patients, but there is a need for further investigation on the effect of PMR on death anxiety.

## 6 Limitations of the Study

The cancer patients had a lot of fears and anxiety due to the disease, and this made cooperation hard for them. We tried to receive their attention by helping them to concentrate more on the PMR technique and explaining the possible benefits of that, but still, it might impact the final results of the study.

The relatively short follow‐up period (1 month) of study, does not allow us for long‐term assessment of PMR's effectiveness. Also, the study's single‐center design limits generalizability. A multi‐center trial would provide more robust conclusions.

Finally, The potential placebo effect, as the intervention group may have experienced benefits simply due to increased attention from researchers.

## Author Contributions

**Hadi Hasani:** investigation, writing – original draft, conceptualization, writing – review and editing. **Majid Kazemi:** supervision, funding acquisition, methodology, formal analysis, writing – review and editing. **Seyed‐Mohammad‐Ebrahim Pourhosseini:** project administration, resources, supervision, methodology, writing – review and editing.

## Consent

Informed consent was obtained from all individual participants included in the study. Research objectives, confidentiality, risks, and potential benefits were presented to participants in online forms. Researchers also provided contact information to support participants in asking questions and to facilitate withdrawal.

## Conflicts of Interest

The authors have no conflict of interest to declare. The supporting source/financial relationships had no involvement in study design; data collection, analysis, and interpretation; writing of the report; or the decision to submit the article for publication.

## Transparency Statement

The lead author, Majid Kazemi, affirms that this manuscript is an honest, accurate, and transparent account of the study being reported; that no important aspects of the study have been omitted; and that any discrepancies from the study as planned (and, if relevant, registered) have been explained.

## Data Availability

Data will be available upon request from the corresponding author.
